# Epicardial left ventricular lead implantation in cardiac resynchronization therapy patients via a video‐assisted thoracoscopic technique: Long‐term outcome

**DOI:** 10.1002/clc.23300

**Published:** 2019-12-14

**Authors:** Massimiliano Marini, Stefano Branzoli, Paolo Moggio, Marta Martin, Giuseppina Belotti, Giulio Molon, Fabrizio Guarracini, Alessio Coser, Silvia Quintarelli, Carlo Pederzolli, Angelo Graffigna, Daniele Penzo, Sergio Valsecchi, Maria Caterina Bottoli, Patrizia Pepi, Roberto Bonmassari, Andrea Droghetti

**Affiliations:** ^1^ Department of Cardiology S. Chiara Hospital Trento Italy; ^2^ Department of Cardiac Surgery S. Chiara Hospital Trento Italy; ^3^ Department of Cardiology Treviglio, ASST Bergamo Ovest Bergamo Italy; ^4^ Department of Cardiology IRCCS Sacro Cuore Don Calabria Hospital Negrar Verona Italy; ^5^ Department of Anesthesiology S. Chiara Hospital Trento Italy; ^6^ CRM Department Boston Scientific Milan Italy; ^7^ Department of Thoracic Surgery ASST Mantova Italy; ^8^ Department of Cardiology ASST Mantova Italy

**Keywords:** CRT, epicardial LV lead implantation, video‐assisted thoracoscopic approach

## Abstract

**Background:**

Epicardial placement of the left ventricular (LV) lead via a video‐assisted thoracoscopic (VAT) approach is an alternative to the standard transvenous technique.

**Hypothesis:**

Long‐term safety and efficacy of VAT and transvenous LV lead implantation are comparable. To test it, we reviewed our experience and we compared the outcomes of patients who underwent implantation with the two techniques.

**Methods:**

The VAT procedure is performed under general anesthesia, with oro‐tracheal intubation and right‐sided ventilation, and requires two 5 mm and one 15 mm thoracoscopic ports. After pericardiotomy at the spot of the epicardial target area, pacing measurements are taken and a spiral screw electrode is anchored at the final pacing site. The electrode is then tunneled to the pectoral pocket and connected to the device.

**Results:**

105 patients were referred to our center for epicardial LV lead implantation. After pre‐operative assessment, 5 patients were excluded because of concomitant conditions precluding surgery. The remaining 100 underwent the procedure. LV lead implantation was successful in all patients (median pacing threshold 0.8 ± 0.5 V, no phrenic nerve stimulation) and cardiac resynchronization therapy was established in all but one patient. The median procedure time was 75 min. During a median follow‐up of 24 months, there were no differences in terms of death, cardiovascular hospitalizations or device‐related complications vs the group of 100 patients who had undergone transvenous implantation. Patients of both groups displayed similar improvements in terms of ventricular reverse remodeling and functional status.

**Conclusions:**

Our VAT approach proved safe and effective, and is a viable alternative in the case of failed transvenous LV implantation.

## 1 INTRODUCTION

Epicardial placement of left ventricular (LV) lead has been proposed as an alternative approach in the case of failure of the transvenous approach during cardiac resynchronization therapy (CRT) device implantation. In our center we developed a minimally invasive video‐assisted thoracoscopic (VAT) technique. We reviewed our experience in order to evaluate the safety and efficacy of this technique. We also assessed long‐term safety and efficacy by comparing the outcome of the study group with that of a control group of patients who underwent standard transvenous LV lead implantation.

## 2 METHODS

### 2.1 Patient selection

The study was approved by the Institutional Review Board and all subjects provided written consent. Patients underwent baseline evaluation, which included demographics and medical history, clinical examination, 12‐lead electrocardiogram, and echocardiogram. Patients were also evaluated by means of a CT scan to rule out any thoraco‐pulmonary disease, spirometry with diffusing capacity of the lung for carbon monoxide, and anesthesiology evaluation. In all patients, previous LV lead implantation had been unsuccessful, owing to unsuitable coronary or subclavian venous anatomy, LV lead dislodgement or failure, phrenic nerve stimulation not correctable by reprogramming, or lead extraction because of infection.

### 2.2 Surgical technique and approach

The technique has been previously described.^1^ In summary, after a general anesthetic had been administered, a double‐lumen endotracheal tube was inserted. With the patient in a right lateral decubitus position (90°) and on single‐lung ventilation, three ports (2 × 5 mm; 1 × 15 mm) were inserted in the left hemithorax. The pericardium was opened posterior to the phrenic nerve. A bare area of the heart, free from visible scarring, in the posterolateral region was visualized between the diagonal and obtuse marginal arteries. A MyoPore sutureless myocardial bipolar pacing lead (Greatbach Medical, New York) was anchored via a thoracoscopic approach by means of the FasTacTM Flex (Greatbatch Medical) steerable lead implantation tool. Finally, the patient was returned to the supine position and the electrode was tunneled to the pectoral pocket and connected to the device.

### 2.3 Patient management

Patients were extubated in the operating room or in the Intensive Care Unit. The chest tube was removed 12 to 24 hours after surgery. No redo surgery was necessary. After discharge, clinic visits were scheduled every 6 months.

### 2.4 Control group

A group of 100 consecutive patients who underwent successful transvenous CRT implantation in our center was used as a control group.

### 2.5 Endpoints

Primary end‐points were: time to death due to any cause and time to the combination of death and cardiovascular hospitalization. Additional endpoints were: device‐related complications, LV ejection fraction and volume change, NYHA class, and ventricular pacing parameters.

### 2.6 Statistical analysis

Descriptive statistics are reported as means ± SD for normally distributed continuous variables, or medians with 25th to 75th percentiles in the case of skewed distribution. Differences between mean data were compared by means of a *T*‐test for Gaussian variables. The Mann‐Whitney test and the Wilcoxon non‐parametric test were used to compare non‐Gaussian variables for independent and paired samples, respectively. Differences in proportions were compared by applying Chi‐square analysis or Fisher's exact test, as appropriate. Event rates were summarized by constructing Kaplan‐Meier curves. The log‐rank test was applied in order to evaluate differences between trends. A *P* < .05 was considered significant for all tests. All statistical analyses were performed by means of SPSS Statistics, software, version 20 (IBM Corp, New York, New York).

## 3 RESULTS

### 3.1 Study population

From January 2008 through February 2017, 105 consecutive patients with standard CRT indications were referred to our center for epicardial LV lead implantation. After pre‐operative assessment, 5 patients were excluded because of concomitant conditions precluding surgery. The remaining 100 patients underwent the surgical procedure.

An additional group of 100 consecutive patients who had undergone successful transvenous LV lead implantation in the period between 2012 and 2015 was analyzed in order to compare long‐term outcomes and event rates. Table 1 shows the clinical characteristics of the two groups.

**Table 1:** Demographics and baseline characteristics of the study population

Characteristics	Thoracoscopic (100)	Transvenous (100)	*P*‐value
Male sex	69 (69%)^a^	80 (80%)^a^	.074^b^
Age (years)	73 (65‐77)^c^	74 (68‐76) ^§^	.578^d^
Ischemic cardiomyopathy	45 (45%)^a^	47 (47%)^a^	.777^b^
Primitive cardiomyopathy	39 (39%)^a^	42 (42%)^a^	.666^b^
NYHA III or IV	69 (69%)^a^	59 (59%)^a^	.141^b^
QRS duration (ms)	160 (155‐190)^c^	160 (135‐173)^c^	.021^d^
LBBB QRS morphology	51 (51%)^a^	62 (62%)^a^	.117^b^
RBBB QRS morphology	6 (6%)^a^	17 (17%)^a^	.015^b^
AF on implantation	28 (28%)^a^	22 (22%)^a^	.327^b^
History of AF	58 (58%)^a^	41 (41%)^a^	.016^b^
Arterial hypertension	70 (70%)^a^	61 (61%)^a^	.181^b^
Diabetes	31 (31%)^a^	32 (32%)^a^	.879^b^
COPD	25 (25%)^a^	13 (13%)^a^	.031^b^
CKD	47 (47%)^a^	25 (25%)^a^	.001^b^
Previous cardiac surgery	35 (35%)^a^	24 (24%)^a^	.088^b^
ACE‐inh/ARB	76 (76%)^a^	70 (70%)^a^	.339^b^
B‐blockers	91 (91%)^a^	85 (85%)^a^	.192^b^
MRA	61 (61%)^a^	57 (57%)^a^	.565^b^
Anticoagulant	53 (53%)^a^	40 (40%)^a^	.065^b^
Antiarrhythmic drug	34 (34%)^a^	18 (18%)^a^	.010^b^
LVEF (%)	28 (21‐32)^c^	28 (25‐33)^c^	.308^d^
LVEDV (mL)	172 (138‐241)^c^	167 (142‐223)^c^	.465^d^
LVESV (mL)	123 (102‐175)^c^	123 (95‐167)^c^	.487^d^
Follow‐up (months)	24 (13–39)^c^	32 (24–46)^c^	.003^d^

Abbreviations: AF, atrial fibrillation; CKD, chronic kidney disease; COPD, chronic obstructive pulmonary disease; LBBB, left bundle branch block; LVEDV, left ventricular end diastolic volume; LVEF, left ventricular ejection fraction; LVESV, left ventricular end systolic volume; MRA, mineralocorticoid receptor antagonists; RBBB, right bundle branch block.

a Number of cases (percentage %).

b Chi‐square test.

c Median (Q1‐Q3).

d Mann‐Whitney *U* test.

In the thoracoscopic group, LV lead implantation was successful in all patients and CRT was successfully established in all but one patient at the end of the procedure. The median pacing threshold was 0.8 V at 0.5 ms (IQR 0.6‐1.2) and no phrenic nerve stimulation was reported at the final electrode position. Fixation in a basal LV segment was achieved in 90 (90%) patients. The median total procedure time was 75 min (IQR 55‐95) and the time to epicardial lead fixation was 30 min (IQR 15‐40). The procedure time was significantly longer in the presence of pleural adherence (127 ± 47 min vs 71 ± 25 min, with vs without, *P* < .001) or pericardial adherence (115 ± 48 min vs 74 ± 30 min, with vs without, *P* = .004). No complications were reported, except for two cases of transitory peri‐electrode bleeding and three cases of ventricular fibrillation induced during the procedure. No sequelae were reported for all these events. During the post‐operative hospital stay, 12 complications occurred in nine patients: five cases of worsening heart failure, one followed by death, four pocket or chest‐wall hematomas (only one requiring intervention), one episode of ventricular tachycardia correctly interrupted by the ICD, one episode of atrial fibrillation, and one dislocation of the right ventricular defibrillation lead, causing inappropriate shocks. The median hospital stay after the procedure was 5 days (IQR 3‐7).

In the transvenous group, the median LV lead pacing threshold was 1.1 V at 0.5 ms. The final position of the LV lead was lateral‐anterolateral. Table 2 compares procedural data of the two groups.

**Table 2:** Pacing threshold parameters and final position of the LV lead

Parameter		Thoracoscopic (100)	Transvenous (100)	*P*‐value
LV lead pacing parameters	Threshold (V at 0.5 ms)	0.8 (0.6‐1.2)^c^	1.1 (0.7‐1.6)^c^	.019^d^
	Impedance (Ω)	612 (503‐707)^c^	999 (683‐1254)^c^	<.001^d^
Position of LV lead tip in LAO view	Posterior	34 (34%)^a^	4 (4%)^a^	<.001^b^
	Postero‐lateral	65 (65%)^a^	9 (9%)^a^	
	Lateral	1 (1%)^a^	43 (43%)^a^	
	Antero‐lateral	0 (0%)^a^	38 (38%)^a^	
	Anterior	0 (0%)^a^	6 (6%)^a^	
Position of LV lead tip in RAO view	Basal	90 (90%)^a^	25 (25%)^a^	<.001^b^
	Mid	9 (9%)^a^	75 (75%)^a^	
	Apical	1 (1%)^a^	0 (0%)^a^	

a Number of cases (percentage).

b Chi‐square test.

c Median (Q1‐Q3).

d Mann‐Whitney *U* test.

### 3.2 Follow‐up

**Figure 1:**
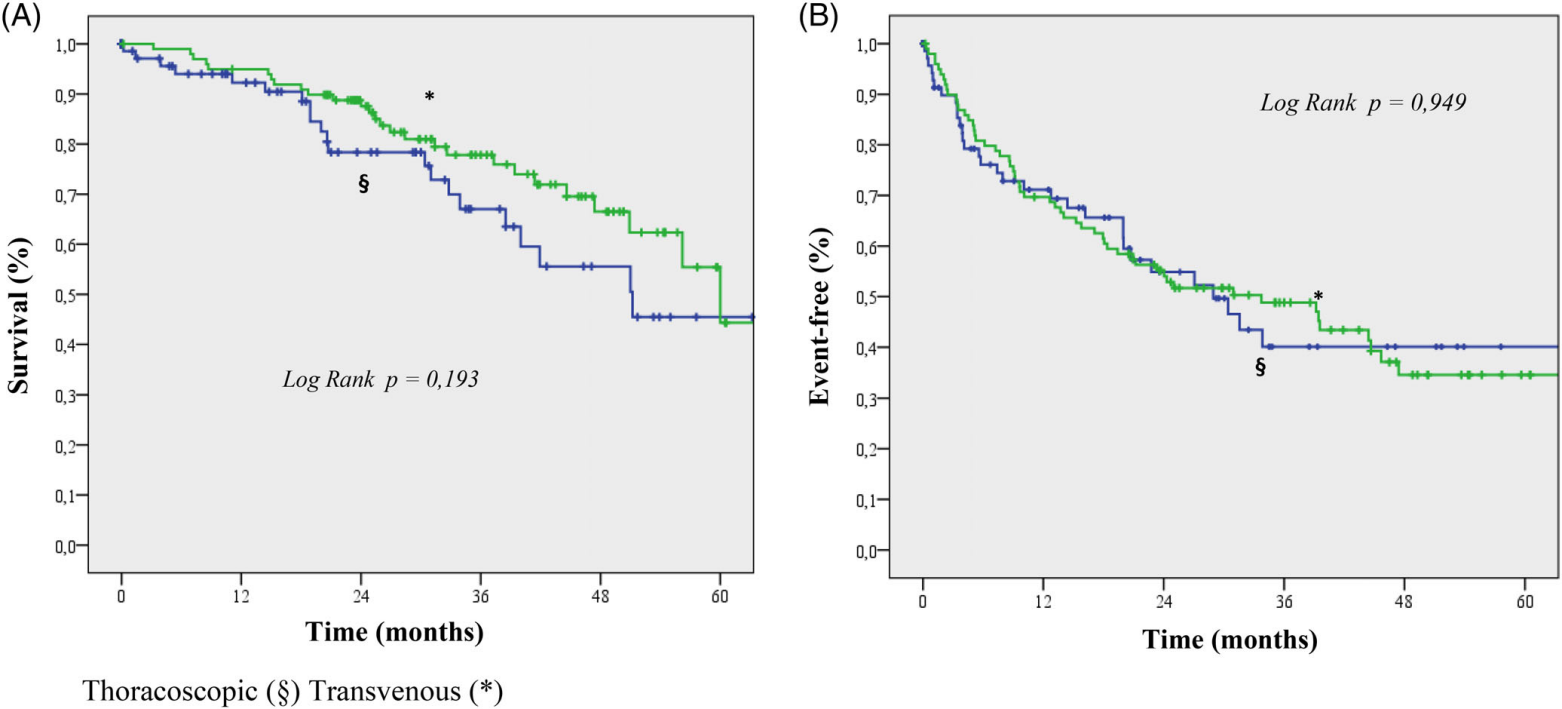
A, Kaplan‐Meier estimate of time to death due to any cause in the thoracoscopic and transvenous groups (log‐rank test, *P* = .193). B, Kaplan‐Meier estimate of time to death due to any cause or cardiovascular hospitalization in the thoracoscopic and transvenous groups (log‐rank test, *P* = .949)

**Figure 2:**
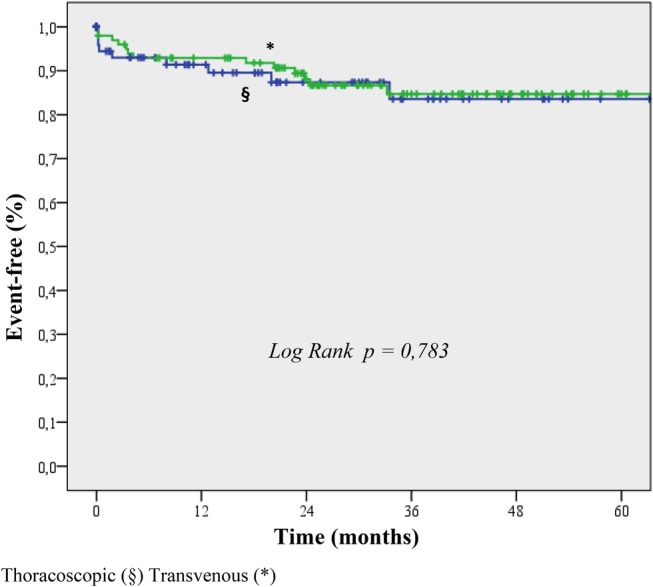
Kaplan‐Meier estimate of time to the first episode of device‐related complication in the thoracoscopic and transvenous groups (log‐rank test, *P* = .783)

In the thoracoscopic group, 21 deaths occurred during a median follow‐up period of 24 months (IQR, 13‐39); in the transvenous group, 27 deaths occurred during a median follow‐up of 32 months (IQR, 24‐46). The rates of death due to any cause were comparable between the groups (Figure 1; log‐rank test, *P* = .193). The rates of death due to any cause and cardiovascular hospitalization were also comparable between the groups (Figure 1; log‐rank test, *P* = .949). Moreover, the risk of device‐related complications was similar between the groups. Figure 2 shows the survival from device‐related complications in both groups (log‐rank test, *P* = .783). In the thoracoscopic group, we recorded five infections (requiring system revision only in one patient), four pocket hematomas (not requiring system revision), one right ventricular lead dislodgement, one system failure, and one replacement of the LV lead. In the transvenous group, six patients had LV lead dislodgement, three had pocket erosion, two had right ventricular lead dislodgement, one had right atrial lead dislodgement, and one system failure.

In both groups, significant reverse remodeling of the LV was observed in terms of increased LV ejection fraction and reduced LV volumes (Table 3). Patients of both groups similarly improved in their functional status, as evidenced by a reduction of patients in NYHA class III‐IV at 1‐year follow‐up. The pacing parameters remained satisfactory in both groups (Table 3).

**Table 3:** Comparison of clinical, echocardiographic, and electrical parameters at baseline and follow‐up in the thoracoscopic and transvenous groups

Impedance	Thoracoscopic (100)			Transvenous (100)			
	Basal	F‐U	*P*‐value	Basal	F‐U	*P*‐value	*P*‐value F‐U
NYHA III or IV	69 (69%)^a^	29 (29%)^a^	<.001^c^	59 (59%)^a^	18 (18%)^a^	<.001^c^	.172^c^
LVEF (%)	30 (22‐33)^d^	36 (31‐43)^d^	<.001^b^	28 (24‐33)^d^	40 (29‐48)^d^	<.001^b^	.550^e^
LVEDV (mL)	180 (140‐224)^d^	170 (115‐224)^d^	.029^b^	167 (143‐226)^d^	152 (123‐183)^d^	.010^b^	.359^e^
LVESV (mL)	117 (105‐160)^d^	104 (64‐160)^d^	.002^b^	124 (94‐167)^d^	88 (63‐122)^d^	<.001^b^	.307^e^
Pacing threshold (V at 0.5 ms)	0.8 (0.6‐1.2)^d^	1.6 (1.1‐2.1)^d^	.002^b^	1.0 (0.6‐1.8)^d^	1.2 (0.6‐2.9)^d^	.100^b^	.326^e^
Impedance (Ω)	645 (483‐705)^d^	383 (344‐430)^d^	<.001^b^	1161 (937‐1272)^d^	669 (486‐913)^d^	.005^b^	<.001^e^

a Number of cases (percentage %).

b Wilcoxon matched‐pairs signed rank test.

c Chi‐square test.

d Median (Q1‐Q3).

e Mann‐Whitney U test.

## 4 DISCUSSION

In clinical practice, the first‐line approach to LV lead implantation during a CRT procedure is the transvenous epicardial approach. The final position of the LV lead depends on the anatomy of the CS, on the performance and stability of the pacing lead and on the absence of phrenic nerve stimulation. Despite all the available technologies, the rate of failed LV lead implantation remains substantial (5‐10%) in transvenous CRT.^2^ Endocardial LV pacing via the trans‐septal route is the only transvenous alternative to the standard technique.^3^ In a recent sub‐analysis from the Alternate Site Cardiac Resynchronization (ALSYNC) study, Biffi et al found that prior non‐responders to CRT were able to improve on LV endocardial pacing via the trans‐septal route.^4^ Many experiences have been published on LV pacing via trans‐septal approach,^3^, ^5^, ^6^, ^7^, ^8^, ^9^, ^10^, ^11^, ^12^, ^13^, ^14^ but no randomized studies have compared its efficacy with that of the epicardial approach.

There are several open‐chest techniques to implanting the LV pacing lead: median sternotomy, fully left thoracotomy, mini‐thoracotomy, video‐assisted thoracoscopy (VAT), and robotically assisted surgery.^15^ These approaches offer the advantages of direct visual control, with the possibility of choosing the lead‐tip position, less fluoroscopy use and the avoidance of intravenous contrast material, whereas the disadvantages are the need for general anesthesia, and the presence of epicardial fat and adhesions.

Two randomized studies have demonstrated the non‐inferiority of open‐chest access vs transvenous approach,^16^, ^17^ in addition to several other non‐randomized studies.^16^, ^17^, ^18^, ^19^, ^20^, ^21^, ^22^, ^23^, ^24^

The VAT technique causes less postoperative pain and requires smaller incisions. The lateral approach enables the target area of the LV (basal postero‐lateral) to be reached more easily than in the supine position previously suggested.^19^ Singh et al^25^ found an association between basal positioning of the LV lead and a lower risk of death and worsening heart failure. In addition, it has been demonstrated that pacing from the site of maximum electrical delay further contributes to improving patient outcomes,^26^ and current guidelines advocate targeting the regions of latest activation.^27^ As the VAT procedure overcomes the limitations of the CS anatomy, it allows easier access to the optimal pacing site, according to the values of pacing parameters and, if possible, to the degree of the electrical delay.

Previous studies have been published on the VAT technique.^19^, ^20^, ^21^, ^24^, ^28^, ^29^, ^30^, ^31^, ^32^, ^33^, ^34^, ^35^, ^36^ We showed that the VAT approach is safe and effective. We included both patients who had undergone unsuccessful *de novo* transvenous LV lead implantation and patients who had responded positively to CRT but had experienced CRT discontinuation due to lead dislodgement.

LV lead implantation was successful in all patients, with few operative complications. The VAT technique resulted in optimal basal postero‐lateral positioning of the electrode, with adequate values of the electrical parameters. Over the long term, the system showed a good safety profile when compared with the transvenous group, with limited complications and stability of the pacing parameters. All‐cause death and cardiovascular hospitalizations were similar. In both groups, there were significant improvements in clinical and echocardiographic parameters, although VAT patients were clinically worse, owing to their comorbidities. Further studies are required in order to determine whether VAT should be limited to patients in whom effective CRT is discontinued, as in the present study, or whether it can also be considered an option for therapy optimization in patients who do not initially improve on CRT.

### 4.1 Study limitations

The main limitation is its lack of randomization. The study group consisted of consecutive patients who were referred to our center for epicardial LV lead implantation. The number of patients who initially underwent an attempt of transvenous implantation at the referring centers is unknown. The control group consisted of consecutive patients who underwent successful transvenous CRT implantation in our center. As data on operative complications in this group were not available for comparison, we limited our comparison to long‐term safety and outcome.

## 5 CONCLUSIONS

In patients in whom transvenous LV lead implantation has previously failed, or in patients who have responded positively to CRT but have experienced CRT discontinuation, the VAT epicardial procedure is a safe and effective technique for LV lead implantation.

## CONFLICT OF INTEREST

S. Valsecchi is a Boston Scientific employee. The other authors declare no potential conflict of interests.
